# Potential added value of a RT-qPCR method of SOX 11 expression, in the context of a multidisciplinary diagnostic assessment of B cell malignancies

**DOI:** 10.1186/s40164-018-0097-6

**Published:** 2018-02-20

**Authors:** Julien Magne, Alizée Jenvrin, Adrien Chauchet, Olivier Casasnovas, Anne Donzel, Laurence Jego, Bernard Aral, Julien Guy, Nathalie Nadal, Dewi Vernerey, Patrick Callier, Francine Garnache-Ottou, Christophe Ferrand

**Affiliations:** 1 INSERM, UMR866, Faculté des Sciences de Santé, Univ. Bourgogne Franche-Comté, 21000 Dijon, France; 2 INSERM, EFS BFC, UMR1098, Univ. Bourgogne Franche-Comté, Besançon, 25000 France; 3 Department of Hematology, University Hospital of Besancon, 25000 Besançon, France; 4 Department of Hematology, University Hospital of Dijon, 21000 Dijon, France; 5 Univ. Bourgogne Franche-Comté, EA 3181, 25000 Besançon, France; 6 Laboratoire de Thérapeutique Immuno-Moléculaire et cellulaire des cancers, INSERM UMR1098, Etablissement Français du Sang Bourgogne/Franche-Comté, 8, rue du Docteur Jean-François-Xavier Girod, 25020 Besançon, France

**Keywords:** Mantle cell lymphoma, SOX11, RT-qPCR-reference genes, ROC curve analysis-threshold

## Abstract

**Background:**

Expression of SRY [sex-determining region Y]-box11 (SOX11) is specific to mantle cell lymphoma (MCL) and contributes, in conjunction with immunoglobulin variable heavy chain gene mutation status, to the identification of two forms of this disease.

**Methods:**

The aim of this report was firstly, to design an easy and suitable RT-qPCR method to quantify SOX11 mRNA expression in mantle cell lymphoma and other B cell malignancies with the proper reference gene; secondly, to define the best threshold of relative quantity of SOX11 mRNA in order to reach the best compromise between sensitivity and specificity.

**Results:**

For best discrimination of MCL and non-MCL groups we determined an area under the curve (AUC) of 0.9750 and a threshold of 1.76 with 100% sensitivity and 88% specificity. AUC and threshold values of respectively 0.91/1.346 [87% sensitivity, 80% specificity] and 0.9525/1.7120 [100% sensitivity, 88% specificity] for GAPDH and RPLP0 respectively denote that the RPLP0 reference gene alone is sufficient for PCR housekeeping gene.

**Conclusion:**

This work describes an RT-qPCR assay for SOX11 expression in order to better characterize MCL at diagnosis. Further studies on larger cohorts are needed to evaluate this molecular tool, especially for the follow-up of minimal residual disease.

**Electronic supplementary material:**

The online version of this article (10.1186/s40164-018-0097-6) contains supplementary material, which is available to authorized users.

## Introduction

Mantle cell lymphoma (MCL) is a subtype of non-Hodgkin lymphoma that represents 5–10% of all lymphomas. It is considered to have a poor prognosis, with a high degree of clonal heterogeneity [1], leading to an overall 5-year survival rate of 50–70% for limited or advanced stages respectively.

If the B cell antigen receptor (BCR) plays a role in MCL lymphomagenesis and disease progression [2], most MCL harbor the t(11;14) (q13;q32) translocation resulting in overexpression of Cyclin D1 (CCND1) mRNA and transcription of the Cyclin D1 nuclear protein. However, transcriptomic studies [3] have revealed MCL cases without CCND1 dysregulation, suggesting that overexpression of CCND1 is not the only factor responsible for the disease. Independently of CCND1 expression, upregulation of SOX11 (SRY [sex-determining region-Y]-box 11), a neural transcription factor, has been detected in all cases of MCL, suggesting it has an important oncogenic role in the development of MCL tumor by regulating PAX5 [4]. SOX11, belonging to the family of genes SOXC, is a transcript factor involved in the embryonic neurogenesis and tissue remodeling, also participating in the control of cell proliferation. It is known that SOX11 associated with WT1 has a synergic effect in the regulation of Wnt-4-promoted nephrogenesis. Several studies in solid tumors (i.e. breast cancer [5]) demonstrate that SOX11, through the expression modulation by epigenetics regulation, plays a transcriptional role that promotes growth and cell differentiation [6]. SOX11 directly binds also to the regulatory regions of PAX5 and BCL6, 2 crucial transcription factors involved in early B-cell development and late differentiation (PAX5) [7].

Absence of SOX11 over-expression in lymphoid progenitor or in mature normal B-cells, correlated with IGHV mutational status in MCL, makes SOX11 a potential biomarker for CCND1-negative MCL, both at diagnosis and in the evaluation of minimal residual disease (MRD). Although the functional role of SOX11 and its prognostic impact on overall survival are still debated, the 2016 revision of the World Health Organization (WHO) classification of lymphoid neoplasms [8] now differentiates two MCL subtypes, with different clinic-pathological manifestations and molecular pathways. The first subtype, with mainly unmutated/minimally mutated IGHV and mostly SOX11-positive, is associated with leukemic non-nodal MCL with bone marrow, splenic and peripheral blood involvement. The second subtype is mostly SOX11-negative, is associated with mutated IGHV and generally indolent, although it may become aggressive by acquiring TP 53 mutation.

For diagnosis purpose, SOX11 expression is primarily analyzed by immunohistochemistry (IHC) or flow cytometry [9]. However, although the availability of the high specificity monoclonal antibody MRQ-58 has improve the diagnosis analysis, the lack of defined cut-off levels of SOX11 expression may generate conflicting results. Thus, molecular biology SOX11 quantification [10], may help to solve and overcome these difficulties but may also be used for MRD quantification.

We aimed to study SOX11 expression with an easy and suitable RT-qPCR method, using appropriate housekeeping genes, and to apply this assay to a cohort of B-cell malignancies, in order to define an easy-to-use molecular tool for distinguishing MCL from other B lymphoproliferative disorders.

## Materials and methods

**Fig. 1:**
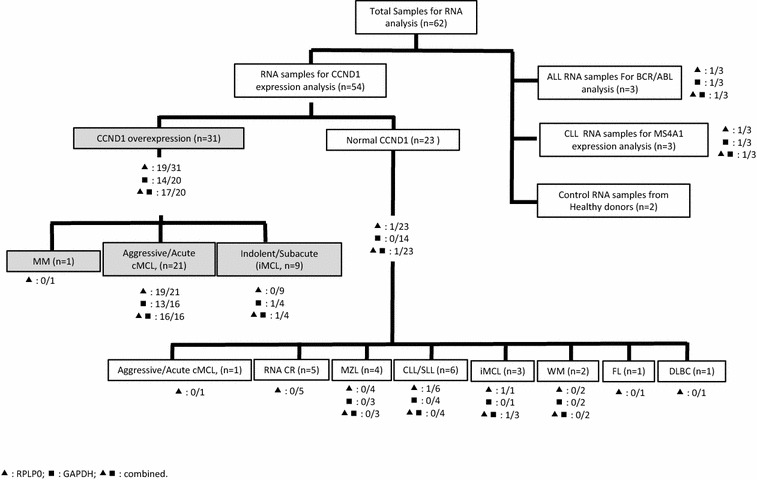
Samples tree screened with SOX11 RTqPCR. Number of patients positive for SOX11 expression respectively with the use of (filled triangle) RPLP0, (filled square) GAPDH or both (filled triangle, filled square) reference genes. Grey boxes represent CCND1 positive samples; CR Complete remission

62 adult patients with (n = 60) or without (n = 2) hematological malignancies were included in the study. Samples were collected from November 2012 to January 2016 at the onco-hematology laboratories of the University Hospitals of Dijon and Besançon, France. 51 samples were collected from routine diagnosis based on semi-quantitative CCND1 expression analysis. MCL diagnosis was established by multidisciplinary analysis, including clinical and paraclinical data, imagery, cytology, pathology, cytogenetics and molecular biology. Among these 54 samples, 31 had CCND1 overexpression, divided between aggressive (cMCL, n = 21), indolent MCL (iMCL, n = 9) and Multiple Myelom (MM, n = 1), whereas the other 23 had Marginal Zone Lymphomas (MZL, n = 4), Waldenström’s Macroglobulinemia (WM, n = 2), Chronic lymphocytic leukemia and small lymphocytic lymphoma (CLL/SLL, n = 4), Follicular lymphoma (FL, n = 1), diffuse large B cell lymphoma (DLBCL, n = 1), (iMCL, n = 3), (cMCL, n = 1), and also RNA samples from patients in complete remission (CR, n = 5) of their hematological malignancies, previously CCND1 positive. The other eight samples were obtained from Chronic Lymphoid Leukemia (CLL, n = 3), Acute Lymphoid Leukemia (ALL, n = 3) or controls (n = 2) (Fig. 1). All samples were obtained after patients provided informed consent, and the study was approved by the local institutional review board.

Mononuclear cells were isolated from the EDTA harvesting tube by Ficoll density Centrifugation. RNAs were extracted using either TRIZOL reagent or RNeasy Mini Kit (Qiagen, Courtaboeuf, France) DNAse exposure was added in order to avoid gDNA contamination. Reverse transcription was performed from RNA. Real time PCR quantification was done in triplicate, in a 20 μl reaction final volume, from 2 μl of cDNA, in a CFX96 thermocycler (Biorad, Marne La Coquette, France) using exonic primer pairs PrimePCR™ PreAmp, Biorad) designed for SYBR^®^ Green gene expression analysis of target SOX11 (Ref #qHsaCED0018676) or housekeeping genes ribosomal protein, large, P0 (RPLP0, ref#qHsaCED0038653) or glyceraldehyde-3-phosphate dehydrogenase (GAPDH, ref #qHsaCED0038674). To improve specificity, a PCR mix containing a Sso7d fusion DNA polymerase (Biorad) was used. Standard two step PCR, according to the manufacturer’s recommendations, was run in a CFX96 thermal cycler (Biorad).

Results were collected and analyzed using the CFX Manager Software (Biorad). Gene expression was assessed using relative quantification with the ΔΔ*CT* method [11] taking into consideration that qPCR efficiencies are equivalent between target and housekeeping gene PCR. Results were retained if at least two replicates had a difference of < 0.4 Ct. PCR efficiencies were assessed by serial log dilutions of a cDNA synthetized from an MCL diagnosis sample in order to generate a standard curve of Ct. Relative expression quantification was calculated against a normal control (calibrator) obtained from either peripheral blood mononuclear cells, or B lymphocyte isolation from healthy donors (n = 3), according to either individual or associated housekeeping genes.

Statistical analyses were performed using R software version 2.15.2 (R Development Core Team; http://www.r-project.org). A p < 0.05 was considered statistically significant and all tests were two-sided.

## Results

The qPCR efficiencies were calculated from the slope of the regression line, plotted from PCR results obtained from Log10 serial dilutions of the same MCL sample. Efficiencies of 98, 103, and 109% for respectively SOX11, GAPDH and RPLP0 PCR were similar according the MiQE guidelines [12] (Additional file 1: Figure S1). For the calibrator (n = 2), an average Ct of 32.6 was obtained for the target SOX11 PCR, whereas we found Ct means of 19.8 and 21.1 respectively for control RPLP0 or GAPDH PCR.

We analyzed typical CCND1 positive MCL samples. For this group, we found a level of relative fold increase (RFI) SOX11 expression of 3352 [min–max: 27–9587.3], 763 [5–4015] or 1070 times [24–2953] higher than within the calibrator, according to RPLP0, GAPDH or both reference genes respectively. Remarkably, one patient with a B-ALL showed a level of SOX 11 expression at a level equal to 209 [0.1–209] (GAPDH), 358 [2–358] (RPLP0), and 273 [0.4–273] (GAPDH & RPLP0) times the control. Interestingly, an IGVH-mutated-CLL patient also had a high level of SOX11 compared to the calibrator: 80 [1–80], 66 [0.2–66], and 72 [0.4–72] respectively for GAPDH, RPLP0 and both housekeeping genes.

Interestingly, all 5 RNA samples harvested from patients in CR of their hematological malignancies and CCND1 positive at diagnosis were categorized at low level of SOX11 expression.

**Fig. 2:**
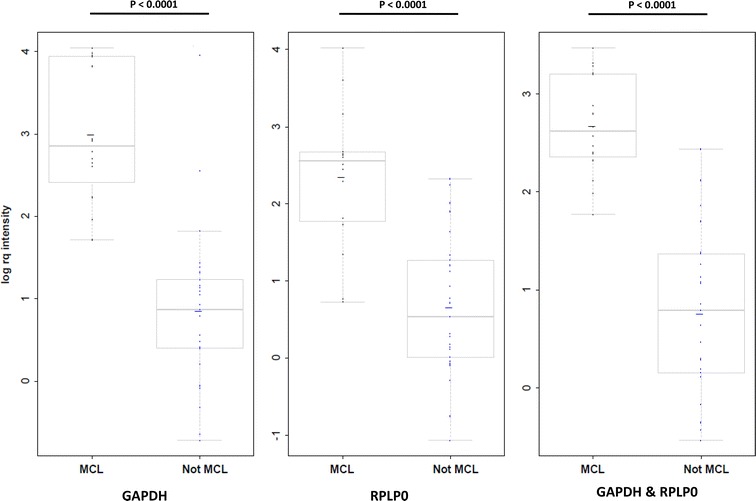
Lor rq intensity according to the different gene combinations are represented as box plots and compared between MCL and other (Non-MCL) groups with the non-parametric Wilcoxon rank sum test. p values are uncorrected for multiple testing but the number of tests (3) and the high level of significance of the results do not require a correction. MCL: Mantle cell lymphoma

Log rq of RFI value was plotted for MCL or “non-MCL” samples for isolated or combined reference genes (Fig. 2). For every reference gene, the difference was highly significant (p < 0.001). Unconditional logistic regression was then performed to model the MCL probability according to the level of log rq gene expressions. A receiver operating characteristic (ROC) curve was constructed (Additional file 1: Figure S2), with calculation of the area under the curve (AUC). The Youden index, representing the difference between the true positive rate and the false positive rate, was maximized to obtain the optimal threshold log rq gene expressions value for the discrimination of MCL and non-MCL groups. For both reference genes, we determined an AUC of 0.9750 and a threshold of 1.76 with 100% sensitivity and 88% specificity. AUC and threshold values of respectively 0.91/1.346 (87% sensitivity, 80% specificity) and 0.9476/1.7120 [100% sensitivity, 88% specificity] for GAPDH and RPLP0 respectively denote that the RPLP0 reference gene alone is sufficient for PCR housekeeping gene.

## Discussion/conclusion

We describe here a simple RT-qPCR protocol that is easy to carry out, to evaluate relative SOX11 expression, and the discriminatory potential of the assay for the diagnosis of MCL disease. We evaluated two different housekeeping genes, either isolated or combined, according to qPCR efficiencies (slopes), in order to apply the ΔΔ*CT* method. As demonstrated, the use of both reference genes GAPDH and RPLP0 allows better discrimination of MCL versus non-MCL samples. However, statistical ROC curve analysis showed that only RPLP0 is required, and is sufficient alone. This will simplify the molecular biology assay and also reduce costs. We also define the best threshold to discriminate MCL versus non-MCL, with a sensitivity of 100% a specificity of 88%.

Despite the low number of samples in our cohort, we detected high level expression in a CLL case. This is in agreement with literature reports of SOX11 expression in 35% of CLL, whereas the remaining 65% lack expression, in association with the IGVH mutational status and aggressiveness of disease [13]. We observed a high level of SOX11 expression in all 3 ALL samples, which has also been reported in the literature for ALL Phi negative case or cell lines. As previously reported by Vegliante et al. [14], SOX11 expression is associated with unmethylated DNA and presence of activating histone marks (H3K9/14Ac and H3K4me3) in some aggressive B-cell neoplasms, whereas it is methylated (with silent histones H3K9me2and H3K27me3) in adult stem cells, normal hematopoietic cells and other lymphoid neoplasms.

In addition to CCND1 expression by RTqPCR, t(11;14) chromosome analysis by FISH or BCL1/JH clonotypic DNA rearrangements by PCR, another method could be useful both to define the disease at diagnosis, for stratification and prognostic value, but also for MRD follow-up. Indeed, MRD quantification is known to be an indicator of treatment response in MCL to prevent clinical relapse [15]. Interestingly, SOX11 expression of RNA samples from patients previously CCND1 positive, are negative at time of CR. Ideally, a multiplex RTqPCR of both CCND1 and SOX11 with 2 appropriate reference genes may enable improved molecular characterization of MCL and follow-up of MRD, for example in order to monitor ibrutinib or lenalidomide treatment efficacy [16]. Association with quantification of CD20 alternative transcripts [17] may be useful, although this molecular tool evaluation has to be properly tested in support of a clinical trial.

For diagnostic purposes, the use of the mantle cell lymphoma international prognostic index (MIPI) in routine clinical practice has shown some limitations, because it imperfectly separates low and intermediate risk groups [18]. Thus, molecular RT-qPCR quantification of SOX11 should add prognostic information to the MIPI, and make it possible to better define patients at risk of poorer outcome who may eligible for alternative treatments, thus minimizing treatment-related morbidity.

In conclusion, this work describes an RT-qPCR assay for SOX11 expression in order to better characterize MCL at diagnosis. Further studies on larger cohorts are needed to evaluate this molecular tool, especially for the follow-up of MRD.

## Additional file

**Additional file 1.** Figures.

## Abbreviations

SOX11: SRY [sex-determining region Y]-box11
RT-qPCR: reverse transcription quantitative polymerase chain reaction
MCL: mantle cell lymphoma
FL: follicular lymphoma
MM: multiple myeloma
DLBCL: diffuse large B cell lymphoma
CCND1: Cyclin D1
MRD: minimal residual disease
WHO: World Health Organization
IGHV: immunoglobulin heavy chain variable
IHC: immunohistochemistry
MZL: marginal zone lymphomas
WM: Waldenström’s Macroglobulinemia
CLL: chronic lymphocytic leukemia
SLL: small lymphocytic lymphoma
ALL: acute lymphoid leukemia
RPLP0: housekeeping genes ribosomal protein large P0
GAPDH: glyceraldehyde-3-phosphate dehydrogenase
cDNA: complementary DNA
gDNA: genomic DNA
ROC: receiver operating characteristic
AUC: area under the curve
MIPI: mantle cell lymphoma international prognostic index
CR: complete remission
