# Supplementary material for: Potential added value of a RT-qPCR method of SOX 11 expression, in the context of a multidisciplinary diagnostic assessment of B cell malignancies
Source: Exp Hematol Oncol. 2018 Feb 20;7:5. doi: 10.1186/s40164-018-0097-6 (PMC5819690; doi:10.1186/s40164-018-0097-6)
Supplement: Supplementary file 1 — Additional file 1. Figures. [file 40164_2018_97_MOESM1_ESM.pptx]

## Slide 1
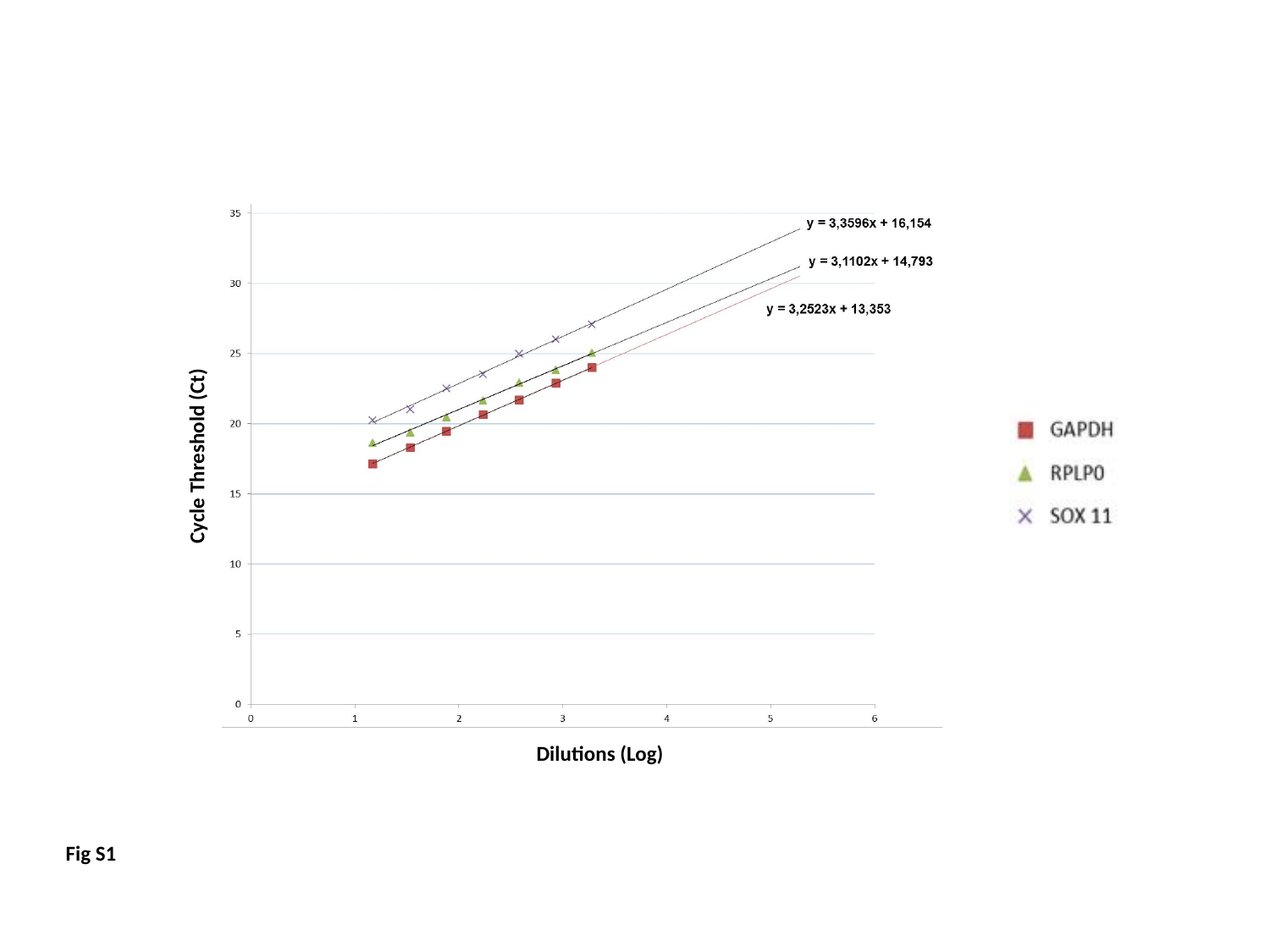

Cycle Threshold (Ct)
Dilutions (Log)
Fig S1
Receiver Operating Characteristic (ROC) curve

## Slide 2
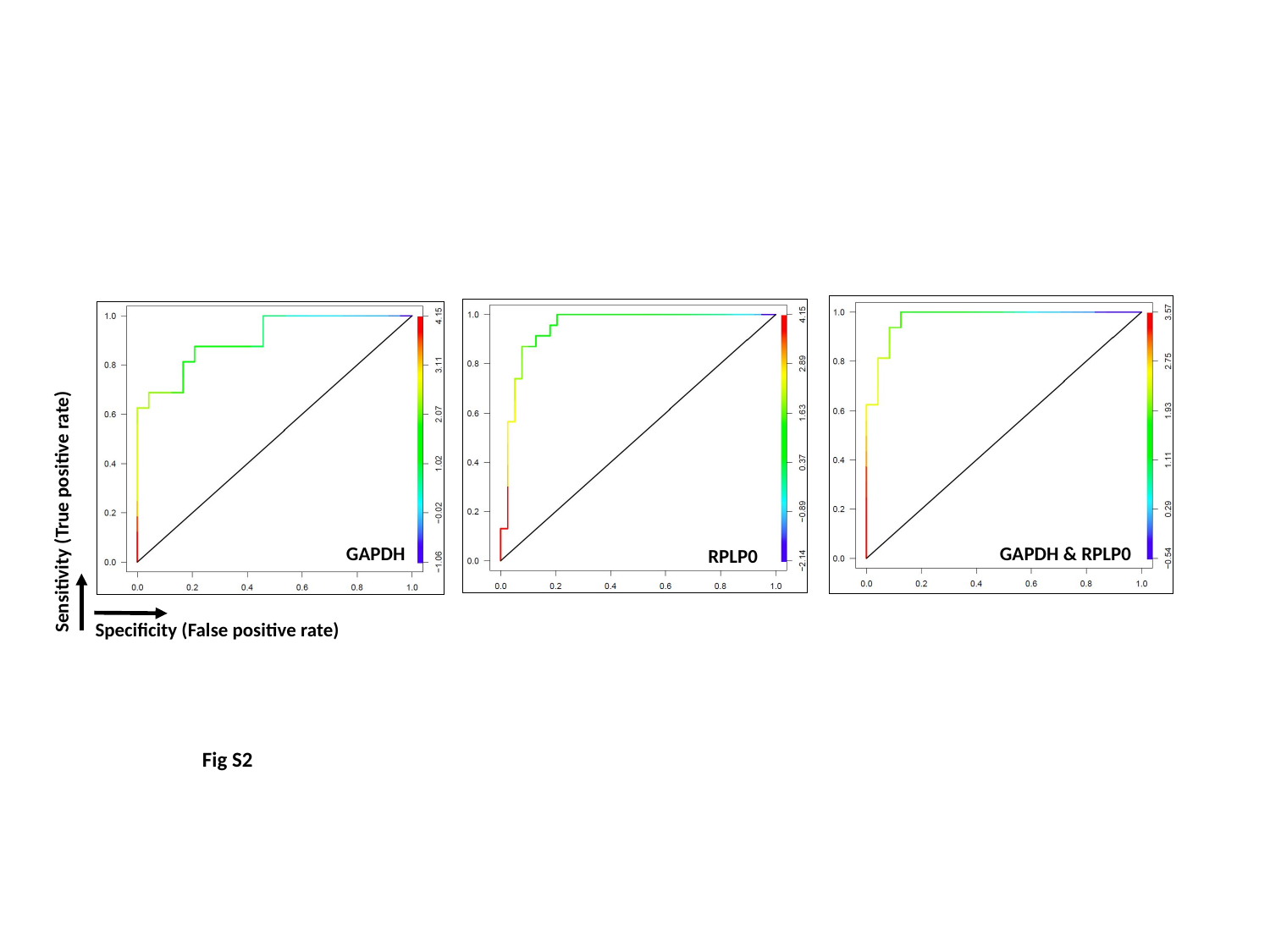

Sensitivity (True positive rate)
GAPDH & RPLP0
GAPDH
RPLP0
Specificity (False positive rate)
Fig S2
Receiver Operating Characteristic (ROC) curve
